# Cytoplasmic-predominant Pten increases microglial activation and synaptic pruning in a murine model with autism-like phenotype

**DOI:** 10.1038/s41380-020-0681-0

**Published:** 2020-02-13

**Authors:** Nicholas Sarn, Ritika Jaini, Stetson Thacker, Hyunpil Lee, Ranjan Dutta, Charis Eng

**Affiliations:** 1 Genomic Medicine Institute, Lerner Research Institute, Cleveland Clinic, Cleveland, OH USA; 2 Department of Genetics and Genome Sciences, Cleveland, OH USA; 3 Germline High Risk Cancer Focus Group, Comprehensive Cancer Center, Case Western Reserve University School of Medicine, Cleveland, OH USA; 4 Cleveland Clinic Lerner College of Medicine, Cleveland, OH 44195 USA; 5 Department of Neurosciences, Lerner Research Institute, Cleveland Clinic, Cleveland, OH USA

**Keywords:** Autism spectrum disorders, Genetics, Neuroscience

## Abstract

Germline mutations in *PTEN* account for ~10% of cases of autism spectrum disorder (ASD) with coincident macrocephaly. To explore the importance of nuclear PTEN in the development of ASD and macrocephaly, we previously generated a mouse model with predominantly cytoplasmic localization of Pten (*Pten*^*m3m4/m3m4*^).Cytoplasmic predominant Pten localization results in a phenotype of extreme macrocephaly and autistic-like traits. Transcriptomic analysis of the *Pten*^*m3m4/m3m4*^ cortex found upregulated gene pathways related to myeloid cell activation, myeloid cell migration, and phagocytosis. These transcriptomic findings were used to direct in vitro assays on *Pten* wild-type and *Pten*^*m3m4/m3m4*^ microglia. We found increased Iba1 and C1q expression with enhanced phagocytic capacity in *Pten*^*m3m4/m3m4*^ microglia, indicating microglial activation. Moreover, through a series of neuron-microglia co-culture experiments, we found *Pten*^*m3m4/m3m4*^ microglia are more efficient at synaptic pruning compared with wild-type controls. In addition, we found evidence for neuron-microglia cross-talk, where *Pten*^*m3m4/m3m4*^ neurons elicit enhanced pruning from innately activated microglia. Subsequent in vivo studies validated our in vitro findings. We observed a concurrent decline in the expression of Pten and synaptic markers in the *Pten*^*m3m4/m3m4*^ cortex. At ~3 weeks of age, with a 50% drop in Pten expression compared with wild-type levels, we observed enhanced activation of microglia in the *Pten*^*m3m4/m3m4*^ brain. Collectively, our data provide evidence that dysregulated Pten in microglia has an etiological role in microglial activation, phagocytosis, and synaptic pruning, creating avenues for future studies on the importance of PTEN in maintaining microglia homeostasis.

## Introduction

The gene encoding Phosphatase and TENsin homolog deleted on chromosome TEN (*PTEN*) is a well-recognized, syndromic risk allele for autism spectrum disorder (ASD), a neurodevelopmental disorder defined by deficits in two core symptom domains: social communication/interaction and restricted/repetitive behavior [1–4]. Carrying a germline *PTEN* mutation is also the molecular criterion for a diagnosis of PTEN Hamartoma Tumor Syndrome (PHTS), irrespective of clinical phenotype. PHTS (OMIM 601728) is an autosomal dominant, cancer predisposition syndrome, where patients present variably but generally have macrocephaly, benign hamartoma of all three germ layers, elevated risks for specific malignancies, and macrocephalic ASD, the latter occurring in about 20% of PHTS individuals [5–7]. Conversely, ~17% of individuals with ASD and macrocephaly harbor a germline *PTEN* mutation [2]. Follow-up studies confirmed this genetic association, adjusting the weighted average for *PTEN* mutation in ASD individuals with coincident macrocephaly to ~10% [4, 8–10]. These genetic data highlight *PTEN* as one of the most prevalent and penetrant ASD-risk genes.

To explore the importance of PTEN in the development of ASD and macrocephaly, we generated a mouse model with a knock-in mutation disrupting two of the four putative nuclear localization signals of Pten [11–13]. The consequent *Pten*^*m3m4*^ mouse is characterized by Pten expression predominantly restricted to the cytoplasm with substantial depletion from the nucleus. This cytoplasmic-predominant model presents with macrocephaly due to megencephaly, hypertrophy of neural somas, gliosis, and autistic-like behavior [12]. RNA sequencing of the *Pten*^*m3m4/m3m4*^ hemi-brain at 2 weeks of age (i.e., P14) and cortex at 6 weeks of age (i.e., P40) demonstrated that the *Pten*^*m3m4/m3m4*^ transcriptome is reflective of idiopathic autism with many of the same genes differentially expressed [13]. Earlier analyses of the transcriptome of the cortex from *Pten*^*m3m4/m3m4*^ mice at 6 weeks of age showed increased expression of genes associated with neuroinflammation and significantly decreased expression of genes associated with synaptic transmission [13].

Our earlier studies on the *Pten*^*m3m4*^ model identified activated microglia in the cortex and hippocampus of the *Pten*^*m3m4/m3m4*^ mouse [12]. Microglia, the resident immune cells of the brain, participate in synaptic pruning, an important neurodevelopmental process. During synaptic pruning, microglia utilize C1q as the regulator of complement-mediated pruning to target immature synapses for engulfment and subsequent removal [14, 15]. It has been demonstrated that altered complement function and C1q expression in microglia are associated with deficits in synaptic transmission and ASD as a consequence of inappropriate synaptic pruning [16–20]. In one such study, the authors show that C1q protein expression is elevated in the serum of children with ASD compared with age-matched controls, further stressing the necessity of proper complement function for neuronal plasticity [16]. There are no published data on the role of Pten as a regulator of microglia function and synaptic pruning. Based on the transcriptomic and preliminary in vivo findings, we hypothesize that constitutive Pten dysfunction predisposes microglia to aberrant activation, leading to exaggerated participation in synaptic pruning during neurodevelopment. Using both in vitro and in vivo models, we demonstrate that Pten activity in microglia is essential for regulating synapse targeting and engulfment, as well as microglial morphology throughout development.

## Materials and methods

### Transcriptomic data analysis

The cortical transcriptome of 6-week-old *Pten*^*m3m4*^ mice (GSE59318) mimics that of idiopathic autism, as described in detail in Tilot et al. [13]. We performed PANTHER GO classification on the list of differentially expressed genes in the cortex of *Pten* homozygous mutant and wild-type mice. Genes annotated as related to biological process and cellular components of interest were identified by immune system processes (GO: 0002376) and the synapse (GO: 0045202), respectively. Next, we performed a “core analysis” using Ingenuity Pathway Analysis (IPA) software (Qiagen, Redwood City, California). The core analysis was mined for significantly enriched pathways relevant to microglia activity, migration, and function, which were used to inform further in vivo and in vitro experiments.

### Animals

Generation and characterization of the *Pten*^*m3m4*^ mouse on a CD1 background has been described previously [12]. The *Pten*^*m3m4*^ mutation is located within exon 7 of *Pten* and consists of five nucleotide substitution mutations, resulting in four nonsynonymous and one synonymous amino acid changes in the third and fourth putative nuclear localization sequences of *Pten* [11, 12]. Genotyping was performed on genomic DNA from clipped toes per the Jackson Laboratory protocol using modified PCR primers. Wild-type allele primers: mPten-F5, 5′-TGGCAGACTCTTCATTTCTGTGGC-3′, and mPten-R6, 5′-ACTTCTTCACAACCACTTCTTTCAAC-3′. Mutant allele primers are mPten-F3, 5′-TACCCGGTAGAATTTCGACGACCT-3′, and mPten-R6, 5′-ACTTCTTCACAACCACTTCTTTCAAC-3′. Mice were maintained on a 14:10 light:dark cycle with access to food and water *ad libitum*. The room temperature was maintained between 18 and 26 °C. Animals were euthanized via CO_2_ asphyxiation followed by cervical dislocation. All experiments were not blinded but randomized and conducted under protocols approved by the Institutional Animal Care and Use Committee (IACUC) at Cleveland Clinic.

We utilized roughly equal numbers of male and female mice for our experiments. We show in a previous study no significant differences between *Pten*^*m3m4/m3m4*^ male and female gross cellular phenotype so all available samples were utilized accordingly [12]. These data are further supported by western blot experiments which denote the sex of each biological replicate used. (Supplementary Figs. 1a–e and 2a, b).

### Western blot analysis

Cortical and hippocampal regions of the brain were isolated, snap-frozen and stored at −80 °C. For making tissue lysates, the tissue was thawed on ice and lysed in RIPA buffer (10 mM Tris-Cl [pH 8], 1 mM EDTA, 0.5 mM EGTA, 1% Triton X-100, 0.1% sodium deoxycholate, 0.1% SDS, 140 mM NaCl, before use add 1 mM PMSF) with phosphatase inhibitor #2 (#P5726-5ML, Sigma, St. Louis, Missouri), phosphatase inhibitor #3 (#P0044-5ML, Sigma) and protease inhibitor (Sigma, #P8345-5ML). All lysates were quantified for protein content using BCA assays, equalized for protein content and 15 μg of protein per sample was loaded on a 4–12% gradient polyacrylamide gel. The separated proteins were transferred to a nitrocellulose membrane, and the membrane was blocked overnight in 2.5% milk diluted in Tris-buffered saline, containing 0.2% Tween-20 (TBST) at 4 °C. Membranes were then washed with TBST and incubated with experiment specific primary antibodies diluted in TBST overnight at 4 °C. The following antibodies were used: Pten (1:5000, #ABM-2025, Cascade Bioscience, Winchester, Massachusetts), Synaptophysin (1:5000, #ab32127, Abcam, Cambridge, Massachusetts), Psd-95 (1:1000, #810401, Biolegend, San Diego, California), and C1q (1:500, #ab71940, Abcam). We removed the primary antibody solution and performed three washes, for 10 min per wash, with TBST. Blots were probed with Goat Anti-Mouse secondary antibody IRDye800CW (1:20,000, #213965, LI-COR, Lincoln, Nebraska) or Goat Anti-Rabbit IRDye680 (LI-COR, #213971) diluted in TBST, for 2 h at room temperature. The membranes were washed three times, 10 min each in TBST and imaged using the Odyssey CLx imaging system (LI-COR). Using ImageJ (National Institute of Health, Bethesda, Maryland, 1995), we performed densitometry analysis on these images to quantify protein expression.

### Cell culture

Mixed glia were obtained by trypsinization of P2 cortices followed by plating on poly-D-lysine coated T-75 culture flasks. Mixed glia cultures, were maintained in DMEM with 10% FBS and 1% Penicillin and Streptomycin (Pen/Strep). Once the mixed glia cultures reached DIV 10, they were agitated at 170 RPM for 1 h in order to isolate primary microglia. Isolated microglia were seeded on poly-D-lysine coated glass cover slips and used for immunofluorescent (IF) staining and phagocytic assays at DIV 3 post-shaking.

We isolated primary neurons from E14.5 cortical tissue. Cortical tissue was mechanically dissociated, trypsinized, and isolated primary neurons were seeded on poly-D-lysine coated cover slips. We grew the neurons in neurobasal media (Thermo-Fisher, #21103–049) supplemented with B27 (1×) (Thermo-Fisher, #17504–044), glutamax (1×) (Thermo-Fisher, #35050061), and 1% Pen/Strep. Neuronal cell cultures were maintained until DIV 14, after which we performed IF staining. If designated for neuron/microglia co-culturing experiments, we isolated microglia via shaking and added them to the neuronal cultures at DIV 7 in a 1:1 ratio (25,000 cells/well in 12-well plate). All mixed glia, primary microglia, and primary neurons were cultured in 5% CO_2_ and 100% humidity at 37 °C. Neurons and microglia remained cultured together in the presence of neurobasal media supplemented with B27 (1×), glutamax (1×), and 1% Pen/Strep to DIV 14, till analysis by IF.

### In vitro immunofluorescent staining

We cultured microglia or neurons on poly-D-lysine (PDL)-coated cover slips until DIV 14. Microglia were washed with ice-cold PBS and fixed in ice-cold methanol for 2 min. This was followed by three washes for 5 min each with ice-cold PBS. We then permeabilized the microglia with 0.03% Triton X-100 dissolved in PBS for 4 min. Next the cultures were blocked with 10% normal goat serum for 1 h at room temperature, followed by incubation with primary antibody: Pten (1:100, #ABM-2025, Cascade Bioscience), Synaptophysin (1:500, #ab32127, Abcam), Iba1 (1:500, #019–19741, Wako, Bellwood, Virginia), Iba1 (1:250, #MABN92, EMD Millipore, Burlington, Massachusetts) or C1q (1:250, #ab71089, Abcam), NeuN (1:500, #MAB377, EDM Millipore), cleaved caspase-3 (1:250, #9664S, Cell Signaling, Danvers, Massachusetts) and Psd-95 (1:250, #810401, Biolegend) diluted in 10% normal goat serum in PBS. We incubated cells in primary antibody overnight at 4 °C. Next, we washed the cells with PBS and then added goat anti-mouse Alexa Fluor 568 secondary antibody (1:2000, #A11031, Thermofisher) and goat anti-rabbit Alexa Fluor 488 (1:2000, #A11008, Thermofisher) diluted in 10% normal goat serum in PBS. The cells were incubated in secondary antibody for 2 h at room temperature (in dark). Post-secondary incubation the cells were washed three times with PBS for 5 min. Finally, the coverslips containing the cells were mounting with Vectashield mounting media with DAPI (Vector Laboratories, Burlingame, California).

### Quantification of functional synapses

We fixed neuron/microglia co-cultures and stained for the synaptic markers, Synaptophysin at 1:500 dilution of primary antibody, and Psd-95 at 1:250 dilution of primary antibody once the neurons had reached DIV 14. Neuron/microglial cultures were imaged using confocal microscopy (Leica Biosystems, Richmond, Ilinois) and analyzed using the Volocity v6.3.0 (Quorum Technologies Inc., Puslinch, Ontario, Canada) to count functional synapses (co-localized Syn and Psd-95) along individual neurites. Functional synapse number was then normalized to the length of the respective neurite.

### Phagocytosis assay

We plated primary microglia at a density of 1 × 10^5^ in a 12-well dish with PDL-coated coverslips for 48 h in a 37 °C cell incubator with 5% CO_2_ and 100% humidity. Next, we blocked 1 μm fluorescent beads (#L1030, Sigma-Aldrich) in FBS for 1 h at 37 °C at a ratio of 1:5 v/v. Florescent beads were diluted with DMEM to reach a final concentration of 0.01% (v/v). Microglial culture media was replaced with 250 μl DMEM containing beads, and incubated for 1 h at 37 °C in a cell incubator. Cultures were washed thoroughly five times with ice-cold PBS and fixed in ice-cold methanol prior to immunofluorescent staining for Iba1 (1:500, #019–19741, Wako).

### Immunofluorescent staining of brain tissue

Mice were euthanized and perfused with ~50 ml of PBS. Brain tissue was extracted and fixed in 4% PFA (pH = 7) for 24 h at 4 °C. PFA was washed three times with PBS, and brain tissue was cryoprotected in 30% sucrose dissolved in PBS for 94 h at 4 °C. 10 μm frozen coronal serial sections were cut on a cryostat and mounted on polarized glass slides (Fisherbrand Superfrost Plus microscope slides, #12–550–15, Fisher Scientific, Waltham, MA). We removed OCT by washing slides in PBS for 10 min. Tissue was permeabilized by incubating the slides with 3% Triton-X dissolved in PBS for 10 min. Slides were washed three times for 5 min each in PBS and probed with experiment specific primary antibodies: Synaptophysin (1:500, #ab32127 Abcam), Iba1 (1:500, #019–19741, Wako), or Iba1 (1:250, #MABN92, EMD Millipore). The slides were incubated overnight at 4 °C in primary antibody, followed by washing PBS and incubation with specific secondary antibodies for 2 h: goat anti-mouse Alexa Fluor 568 (1:2000, #A11031, Thermofisher) and goat anti-rabbit Alexa Fluor 488 (1:2000, #A11008, Thermofisher). Post incubation, slides were washed and mounted in Vectashield medium with DAPI (Vector Laboratories), coverslipped, and sealed with nail polish.

### Immunofluorescent quantification

We captured images of brain sections as well as cells grown in vitro as confocal images using a Leica TCS-SP8-AOBS inverted confocal microscope (Leica Microsystems, GmbH, Wetzlar, Germany). Brain section, primary neuron, and/or microglial co-cultures were imaged with a minimum of *n* = 5 biological replicates. We used ImageJ software to measure area and intensity of the stain and calculated integrated density of brain images. In addition, ImageJ was used to measure area of stain per microglia in vitro and in vivo to assess morphological changes beyond “bushy” and “amoeboid” [21]. Primary neuron/microglial co-cultures were analyzed using the Volocity 3D imaging software in order to count the number of functional synapses and normalize these values to neurite length.

Due to the low abundance of primary microglia resulting from our isolation protocol, we pooled microglia from various cultures where appropriate. These microglia were maintained in culture and stained accordingly. We captured a minimum of five images per cultured microglia genotype. Finally, we quantified these images using ImageJ to measure signal, area and integrated density.

### Statistical analysis

Sample size was determined according to our previous studies utilizing the *Pten*^*m3m4*^ model [12, 13, 22]. These studies were conducted in a non-blinded manner with sample utilization being randomized. Statistical tests are justified according to the distribution of the data, and also if data meets the assumptions of each analysis. We analyzed normally distributed data using a one-way analysis of variance (ANOVA) or Student’s t-test, where appropriate (Graph Pad Prism 8). After performing a one-way or two-way ANOVA when appropriate, we performed a post-hoc Tukey–Kramer analysis (F). When data were not normally distributed, we performed non-parametric analyses including Mann–Whitney U and Kruskall–Wallis tests (H), where appropriate (Graph Pad Prism 8). *P* values that are less than 0.05 were considered statistically significant. We calculated effect sizes as the mean difference with 95% confidence intervals using the R package DABESTR [23]. In addition, Spearman rho R correlation matrix was used to determine correlations between data plots when appropriate (Graph Pad Prism 8).

## Results

### The *Pten*^*m3m4*^ neural transcriptome is enriched in neuroinflammation networks, including microglial activation and phagocytic activity

Our earlier studies have revealed a neuroinflammatory signature in the cortical transcriptome of 6-week-old *Pten*^*m3m4/m3m4*^ mice [4]. Using this platform, we found a significant increase in expression of complement-related proteins, and genes implicated in synaptic pruning in *Pten*^*m3m4/m3m4*^ mice compared with *Pten*^*WT/WT*^ controls (Table 1). In addition, numerous inflammation-related and synapse-related genes also showed differential expression (Supplementary Tables 1 and 2). These results highlight dysregulation of mediators of immune and synaptic function in *Pten*^*m3m4/m3m4*^ mice.

**Table 1:** Expression of complement-related genes in the cortex of *Pten*^*m3m4/m3m4*^ compared with wildtype.

Gene ID	Fold change	*P v*alue	*Q* value
*C1qa*	2.40	5.00E−05	0.00191
*C1qb*	2.36	5.00E−05	0.00191
*C1qc*	2.40	5.00E−05	0.00191
*C3ar1*	2.40	5.00E−05	0.00191
*Cx3Cr1*	1.51	5.00E−05	0.00191
*Itgam*	1.63	5.00E−05	0.00191
*Itgb2*	1.99	5.00E−05	0.00191
*Trem2*	1.75	5.00E−05	0.00191

**Fig. 1:**
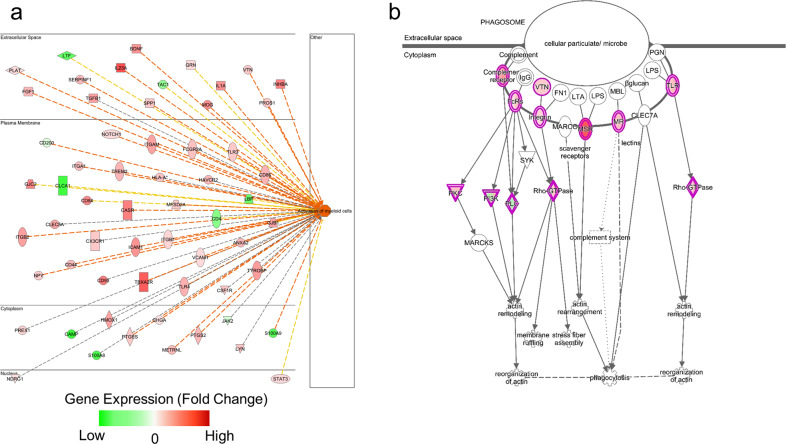
Ingenuity pathway analysis of *Pten*^*m3m4/m3m4*^ cortical transcriptome data. **a** Increased “Activation of Myeloid Cells” (60 genes; *p* value = 1.38E−10; z-score = 3.36) pathway enriched in the *Pten*^*m3m4/m3m4*^ cortex compared with the *Pten*^*WT/WT*^ cortex at 6 weeks of age. **b** Differential expression data overlaid on enriched “Phagosome Formation” (IPA) canonical pathway for *Pten*^*m3m4/m3m4*^ vs. *Pten*^*WT/WT*^ comparison.

Subsequent gene enrichment and pathway analyses (Ingenuity Pathway Analysis [IPA] software) on the transcriptomic data from the cortices of 6-week-old *Pten*^*m3m4/m3m4*^ mice, identified significant enrichment of pathways defined as “activation of myeloid cells”, “phagocytosis”, “cell movement of phagocytes” (Fig. 1a, Supplementary Fig. 3a, b) and phagosome formation (Fig. 1b). Collectively, these analyses strongly suggest activation of microglia and increased phagocytosis.

### *Pten*^*m3m4/m3m4*^ primary microglia in culture have cytoplasmic predominant Pten localization

**Fig. 2:**
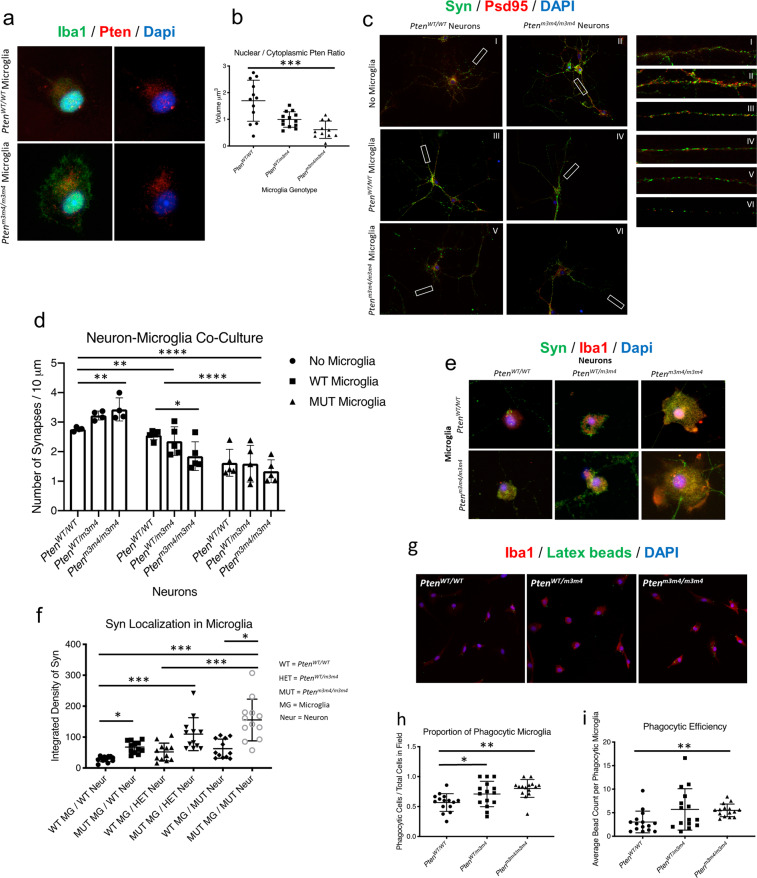
Cytoplasmic predominant Pten localization results in increased microglia-dependent synaptic pruning and phagocytosis in vitro. **a** Immunofluorescent co-staining of Iba1 (green) and Pten (red) in *Pten*^*WT/WT*^ and *Pten*^*m3m4/m3m4*^ primary microglia. **b** Quantification of nuclear and cytoplasmic distribution of Pten within *Pten*^*WT/WT*^ (mean volume = 1.7 ± 0.77 μm^3^; *H* = 14.1) and *Pten*^*m3m4/m3m4*^ (mean volume = 0.61 ± 0.32 μm^3^; *H* = 14.1, *p* value = 0.0005). **c** Immunofluorescent co-staining of Syn (green) and Psd-96 (Red) in neuron-microglia co-cultures. **d** Quantification of functional synapse number along 10 µm of neurite length of each neuron-microglia co-culture shows microglia account for 68.57% of the observable interaction across all neuronal conditions irrespective of neuronal genotype (*p* value ≤ 0.0001, *F* = 52.57): *Pten*^*m3m4/m3m4*^ microglia compared with no microglia (mean difference in synapses per 10 µm = 1.62 ± 1.23 to 2.01; *F* = 52.57, *p* value < 0.0001) and *Pten*^*m3m4/m3m4*^ microglia compared with *Pten*^*WT/WT*^ microglia (mean difference in synapses per 10 µm = 0.74 ± 0.37 to 1.10; *F* = 52.57, *p* value < 0.0001) and *Pten*^*WT/WT*^ microglia compared with no microglia (mean difference in synapses per 10 µm = 0.88 ± 0.49 to 1.27; *F* = 52.57, *p* value < 0.0001). In addition, *Pten*^*m3m4/m3m4*^ neurons (mean synapses per 10 µm = 1.3 ± 0.4 vs. 3.2 ± 0.2; *F* = 28, *p* value < 0.0001) compared with *Pten*^*WT/m3m4*^ neurons (mean synapses per 10 µm = 1.6 ± 0.6 vs. 3.2 ± 0.2; *F* = 13, *p* value = 0.001) and *Pten*^*WT/WT*^ neurons (mean synapses per 10 µm = 1.6 ± 0.5 vs. 2.8 ± 0.07; *F* = 20, *p* value = 0.0004). **e** Immunofluorescent co-staining in neuron-microglia co-cultures shows Syn (green) and Iba1 (red). **f** Quantification of Syn localization within microglia co-cultured with primary neurons (mean Syn integrated density: *Pten*^*m3m4/m3m4*^ microglia with *Pten*^*WT/WT*^ neurons = 67.4 ± 19.1; *H* = 42.0, *p* value = 0.04; *Pten*^*WT/WT*^ microglia with *Pten*^*WT/WT*^ neurons = 29.1 ± 9.0; *H* = 42.0; *Pten*^*m3m4/m3m4*^ microglia with *Pten*^*m3m4/m3m4*^ neurons = 155.4 ± 67.4; *H* = 42.0, *p* value < 0.0001; *Pten*^*WT/WT*^ microglia with *Pten*^*m3m4/m3m4*^ neurons = 62.5 ± 30.1; *H* = 42.0). **g** Phagocytosis assay with primary microglia cultures co-stained for Iba1 (red) and fluorescent latex beads (green). **h**, **i** Phagocytosis assay quantifications of phagocytic microglia (mean phagocytic microglia in observed field: *Pten*^*WT/WT* ^= 0.6 ± 0.15; *H* = 12.69; *Pten*^*WT/m3m4*^ microglia = 0.78 ± 0.3; *H* = 12.69, *p* value = 0.03; *Pten*^*m3m4/m3m4*^ microglia = 0.8 ± 0.15; *H* = 12.69, *p* value = 0.001) and phagocytic ability of microglia to uptake fluorescent beads (mean phagocytized beads: *Pten*^*WT/W* ^= 3.0 ± 2.3; *H* = 10.5; *Pten*^*m3m4/m3m4*^ = 5.5 ± 1.3; *H* = 10.5, *p* value = 0.005).

To evaluate Pten localization in mice with *Pten*^*m3m4*^ mutations, primary microglia from *Pten*^*WT/WT*^*, Pten*^*WT/m3m4*^*, and Pten*^*m3m4/m3m4*^ mice were co-stained for Pten and Iba1. 3D rendering of the microglia showed Pten localization significantly increased in the cytoplasm compared with the nucleus of *Pten*^*m3m4/m3m4*^ microglia (mean Pten volume: *Pten*^*WT/WT*^ = 1.7 ± 0.77 μm^3^ vs. *Pten*^*m3m4/m3m4* ^= 0.61 ± 0.32 μm^3^; *p* value = 0.0005) (Fig. 2a, b).

### *Pten*^*m3m4/m3m4*^ microglia show evidence of enhanced synaptic pruning activity

To evaluate synaptic pruning capacity of *Pten* mutant microglia we co-cultured primary neurons and microglia from *Pten*^*WT/WT*^*, Pten*^*WT/m3m4*^, or *Pten*^*m3m4/m3m4*^ mice in differing combinations for 72 h. Co-localization of pre- and post-synaptic markers, Syn and Psd-95, respectively, was used to determine presence of functional synapse and normalized to neurite length (Fig. 2c, d). Upon analysis of our microglia-neuron co-cultures we found the most dramatic decrease in synapse number with *Pten*^*m3m4/m3m4*^ microglia co-cultures compared with *Pten*^*WT/WT*^ microglia co-cultures (mean difference of synapses per 10 µm = 0.74; *F* = 57.52, *p* value ≤ 0.0001) and also compared with neuronal cultures without microglia (mean difference of synapses per 10 µm = 1.62; *F* = 57.52, *p* value ≤ 0.0001) (Fig. 2c, d). Subsequent analysis shows the highest numbers of functional synapses per 10 µm of neurite length were observed when no microglia were present in the neuronal cultures of all genotypes (mean synapses per 10 µm*: Pten*^*WT/WT* ^= 2.8 ± 0.07; *Pten*^*WT/m3m4* ^= 3.2 ± 0.2; *Pten*^*m3m4/m3m4* ^= 3.4 ± 0.4). *Pten*^*m3m4/m3m4*^ neurons show significantly more synapses compared with *Pten*^*WT/WT*^ neurons (mean difference of synapses per 10 µm = 0.6; *F* = 7.865, *p* value = 0.0095) (Fig. 2c, d and Supplementary Fig. 4a, b, Supplementary Table 3). In microglia co-cultures the number of synapses were substantially reduced compared with neuronal mono-cultures. The largest decrease in synapse number was observed when *Pten*^*m3m4/m3m4*^ neurons were cultured with *Pten*^*m3m4/m3m4*^ microglia (mean synapses per 10 µm = 1.3 ± 0.4) compared with *Pten*^*WT/m3m4*^ neurons (1.6 ± 0.6) and *Pten*^*WT/WT*^ neurons (1.6 ± 0.5) (Fig. 2c, d and Supplementary Fig. 4c–e, Supplementary Table 3). Interestingly, even in *Pten*^*WT/WT*^ microglia co-cultures, the largest decrease was seen with *Pten*^*m3m4/m3m4*^ neurons (Fig. 2c, d and Supplementary Fig. 4c, d). We confirmed that the decrease in synapse number in the co-culture was due to microglial- pruning, and not due to neuronal death by co-staining for NeuN and Cleaved Caspase-3. No increase in neuronal death was observed (Supplementary Fig. 5a). These data show that neurons from *Pten*^*m3m4/m3m4*^ mutants form more synapses compared with *Pten*^*WT/WT*^ neurons as shown in earlier studies. However, in a systemic germline *Pten*^*m3m4/m3m4*^ mutant system, our data suggest cross-talk between neurons and microglia, resulting in significantly increased synaptic pruning in *Pten*^*m3m4/m3m4*^ mutants compared with *Pten*^*WT/WT*^ mice.

### *Pten*^*m3m4/m3m4*^ microglia have enhanced ability for synaptic engulfment and phagocytosis

To further confirm that *Pten*^*m3m4/m3m4*^ microglia have enhanced pruning efficiency, we used the microglia and neuronal co-culture system. We observed increased synaptic engulfment in *Pten*^*m3m4/m3m4*^ microglia compared with *Pten*^*WT/WT*^ microglia when co-cultured with *Pten*^*WT/WT*^ neurons (Fig. 2e, f). Interestingly, when *Pten*^*m3m4/m3m4*^ neurons were present in the co-culture system, there was an observed increase in synaptic pruning by both *Pten*^*m3m4/m3m4*^ and *Pten*^*WT/WT*^ microglia. The highest numbers of engulfed synapses were observed in *Pten*^*m3m4/m3m4*^ microglia and *Pten*^*m3m4/m3m4*^ neuron co-cultures (Fig. 2e, f, and Supplementary Table 4). Bead-based phagocytosis assays revealed significantly increased numbers of beads per microglia (*Pten*^*WT/WT* ^= 3.0 ± 2.3 vs. *Pten*^*m3m4/m3m4*^ = 5.5 ± 1.3; *p* value = 0.005), as well as increased numbers of phagocytic microglia in cultures of *Pten*^*m3m4/m3m4*^ microglia vs. *Pten*^*WT/WT*^ microglia (*Pten*^*m3m4/m3m4*^ microglia = 0.8 ± 0.15 vs. *Pten*^*WT/WT* ^= 0.6 ± 0.15; *p* value = 0.001) (Fig. 2g–i). Collectively, these data imply that *Pten*^*m3m4/m3m4*^ microglia have a higher capacity to engulf synapses, and that cross-talk via *Pten*^*m3m4/m3m4*^ neurons may enhance pruning when co-cultured together regardless of microglia genotype.

### Enhanced synaptic pruning by *Pten*^*m3m4/m3m4*^ microglia is associated with increased C1q production and deposition

**Fig. 3:**
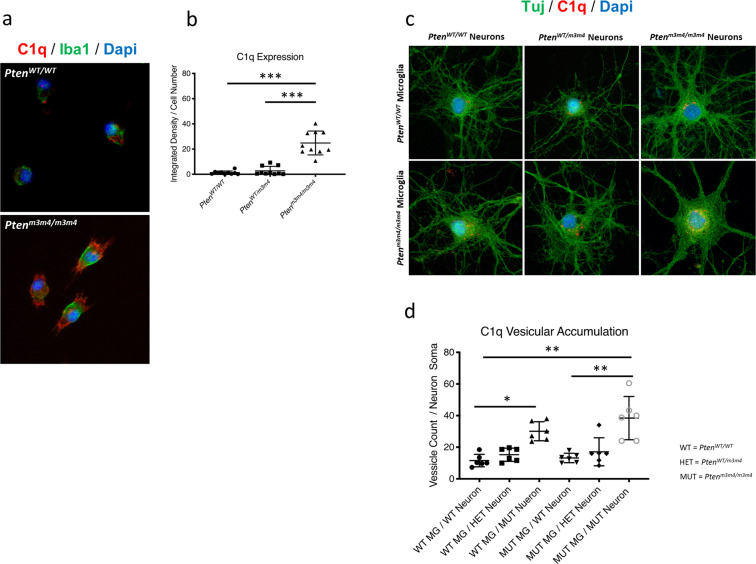
Increased expression of C1q in *Pten*^*m3m4/m3m4*^ microglia and enhanced C1q accumulation on synapses of *Pten*^*m3m4/m3m4*^ neurons. **a** Immunofluorescent staining for C1q (red) and Iba1 (green) in primary microglia. **b** Quantification of C1q expression in primary microglia (mean integrated density of C1q: *Pten*^*WT/WT* ^= 1.3 ± 1.4; *H* = 19.45; *Pten*^*WT/m3m4* ^= 2.8 ± 0.56; *H* = 19.45, *p* value = 0.0008; *Pten*^*m3m4/m3m4*^ = 24.8 ± 9.5; *H* = 19.45, *p* value = 0.0002). **c** Immunofluorescent co-staining for C1q (red) and Tuj1 (green) in neuron-microglia co-cultures. **d** Quantification of C1q foci on neuronal somas (mean C1q foci number: *Pten*^*m3m4/m3m4*^ neurons with *Pten*^*WT/WT*^ microglia = 30.1 ± 6.0; *H* = 23.64, *p* value = 0.01; *Pten*^*m3m4*^ neurons with *Pten*^*m3m4/m3m4*^ microglia = 38.4 ± 13.6; *H* = 23.64, *p* value = 0.02; *Pten*^*WT/WT*^ neuron with *Pten*^*WT/WT*^ microglia = 11.6 ± 3.9; *Pten*^*WT/WT*^ neuron with *Pten*^*m3m4/m3m4*^ microglia = 13.2 ± 3.0).

Since complement expression in primary microglia is the key regulator of microglial-dependent synaptic pruning [17–19], we examined C1q protein levels. RNA-seq and ELISA analyses on cortical lysates as well as IF staining showed increases in C1q expression in *Pten*^*WT/m3m4*^ and *Pten*^*m3m4/m3m4*^ microglia (C1q integrated density: *Pten*^*WT/WT* ^= 1.3 ± 1.4; vs. *Pten*^*m3m4/m3m4*^ = 24.8 ± 9.5, *p* value = 0.0002) (Fig. 3a, b; Supplementary Fig. 5b). Next, we co-labeled neurons with class III β-tubulin (Tuj1) and C1q. In comparison to *Pten*^*WT/WT*^ neurons, *Pten*^*m3m4/m3m4*^ neurons showed significantly increased C1q foci present on their soma when co-cultured with either of the *Pten*^*WT/WT*^ or *Pten*^*m3m4/m3m4*^ microglia (C1q foci *Pten*^*m3m4/m3m4*^ neurons: *Pten*^*WT/WT*^ microglia = 30.1 ± 6.0, *p* value = 0.01 vs. *Pten*^*m3m4/m3m4*^ microglia = 38.4 ± 13.6, *p* value = 0.02) (Fig. 3c, d). These data suggest that *Pten*^*m3m4/m3m4*^ microglia are independently predisposed to enhanced synaptic pruning via increased expression of C1q. In addition, the microglial-neuron cross-talk, specifically that involving *Pten*^*m3m4/m3m4*^ neurons, is able to enhance microglial pruning efficiency irrespective of microglial genotype likely resulting in a “double whammy” for over-pruning of synapses in *Pten*^*m3m4/m3m4*^ mice.

### *Pten*^*m3m4/m3m4*^ mice have reduced expression of synaptic markers and increased microglia activation concurrent with decreased Pten expression in the forebrain

**Fig. 4:**
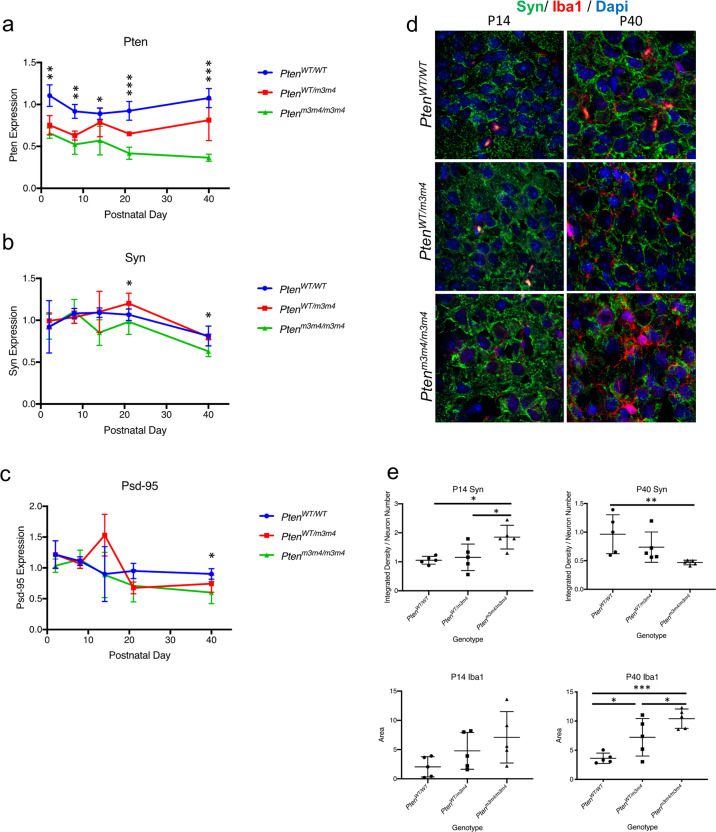
Dysregulated Pten expression in forebrains of *Pten*^*m3m4/m3m4*^ mice correlates with decreases in synaptic marker expression and increased microglial activation in vivo. **a** Expression of Pten in the forebrain of *Pten*^*WT/WT*^, *Pten*^*WT/m3m4*^, and *Pten*^*m3m4/m3m4*^ mice quantified by western blot analyses at P2, P8, P14, P21, and P40; P2 (relative expression = 0.63; *H* = 11.92, *p* value = 0.002), P8 (relative expression = 0.52; *H* = 11.70, *p* value = 0.002), P14 (relative expression = 0.69; *H* = 7.98, *p* value = 0.01), P21 (relative expression = 0.41; *H* = 14.24, *p* value = 0.0005), P40 (relative expression = 0.36; *H* = 12.44, *p* value = 0.001). **b** Expression of Syn in the forebrain of *Pten*^*WT/WT*^, *Pten*^*WT/m3m4*^, and *Pten*^*m3m4/m3m4*^ mice quantified by western blot analyses at P2, P8, P14, P21, and P40; P40 (relative expression = 0.62; *H* = 10.43, *p* value = 0.02). **c** Expression of Psd-95 in the forebrain of *Pten*^*WT/WT*^, *Pten*^*WT/m3m4*^, and *Pten*^*m3m4/m3m4*^ mice quantified by western blot analyses at P2, P8, P14, P21, and P40; P40 (relative expression = 0.60; *H* = 8.6, *p* value = 0.01). **d** Immunofluorescent staining for Syn (green) and Iba1 (red) in *Pten*^*WT/WT*^, *Pten*^*WT/m3m4*^, and *Pten*^*m3m4/m3m4*^ CA3 hippocampal regions at P14 and P40. **e** Quantification of Syn in CA3 region of the hippocampus at P14 (mean integrated density of Syn: *Pten*^*WT/WT* ^= 1.06 ± 0.13; *Pten*^*WT/m3m4*^ = 1.15 ± 0.46; *F* = 7.17, *p* value = 0.02; *Pten*^*m3m4/m3m4* ^= 1.85 ± 0.41; *F* = 7.17, *p* value = 0.01) and P40 (mean integrated density Syn: *Pten*^*WT/WT*^ = 0.96 ± 0.33; *Pten*^*m3m4/m3m*^ = 0.47 ± 0.04; *F* = 4.9, *p* value = 0.03), as well as microglia cell area P40 (mean Iba1 area:  *Pten*^*WT/WT*^ = 3.62 ± 0.89; *Pten*^*m3m4/m3m4*^ = 10.41 ± 1.70; *F* = 12.5, *p* value = 0.0009).

**Fig. 5:**
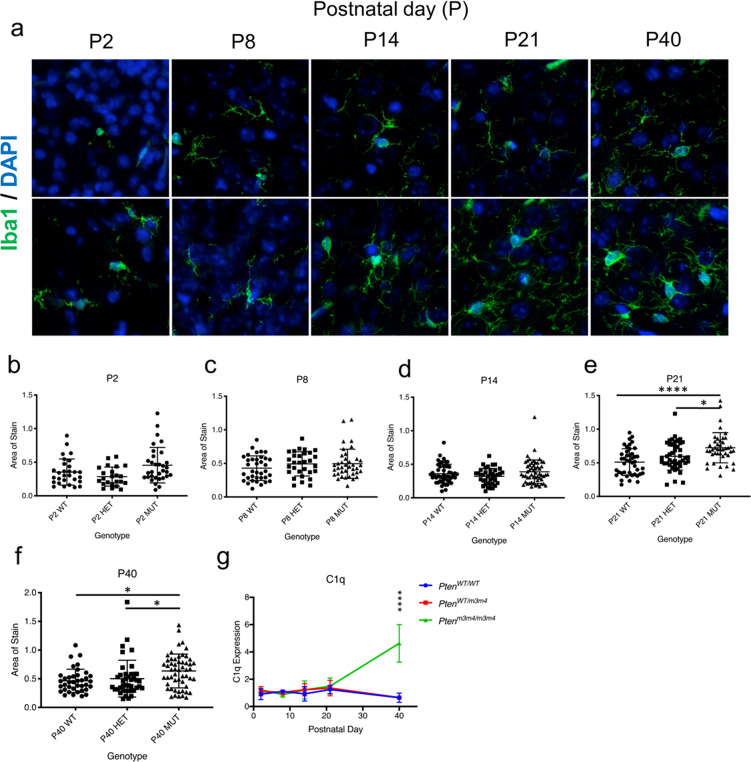
Increased microglial activation and C1q expression in cortex of *Pten*^*m3m4/m3m4*^ mice. **a** Immunofluorescence staining of Iba1 (green) to examine cell morphology in microglial cultures derived from *Pten*^*WT/WT*^*, Pten*^*WT/m3m4*^, and *Pten*^*m3m/m3m4*^ cortex tissue developmental stages P2, P8, P14, P21, and P40. **b**–**f** Quantification of Iba1 stain area in *Pten*^*WT/WT*^*, Pten*^*WT/m3m4*^, and *Pten*^*m3m/m3m4*^ cortical microglia at P2, P8, P14, P21, and P40. **g** Expression of C1q in the forebrain of *Pten*^*WT/WT*^, *Pten*^*WT/m3m4*^, and *Pten*^*m3m4/m3m4*^ mice quantified by western blot analyses at P2, P8, P14, P21, and P40, (P40 *Pten*^*m3m4/m3m4*^ relative expression = 4.6 ± 1.4; *H* = 11.01, *p* value = 0.009).

The in vitro data presented above show that *Pten*^*m3m4/m3m4*^ microglia have enhanced phagocytosis and synaptic pruning capacity with increased C1q expression. These findings were further validated by our *Pten*^*m3m4/m3m4*^ murine model characterized by a systemic cytoplasmic predominant Pten. Western blot analyses showed that Pten expression is significantly reduced in the hemibrains of *Pten*^*m3m4/m3m4*^ mice compared with those of *Pten*^*WT/WT*^ controls at P2 (relative expression = 0.63, *p* value = 0.002) and P8 (relative expression = 0.52, *p* value = 0.002) (Fig. 4a–c) and later ages (Supplementary Fig. 1a–e). As the *Pten*^*m3m4/m3m4*^ mouse continues to age, Pten expression progressively declines in the cortex until reaching its nadir at P40 (relative expression = 0.36, *p* value = 0.001) (Fig. 4a). Interestingly, it is also at this time point, the terminal age for this mouse model, we observe a significant decrease in expression of Syn (relative expression = 0.62, *p* value = 0.02) and Psd-95 (relative expression = 0.60, *p* value = 0.01) (Fig. 4a–c). This is concordant with increased expression of C1q (relative expression = 4.6, *p* value = 0.001) (Fig. 5g) in the *Pten*^*m3m4/m3m4*^ cortex. In order to determine if a correlation existed between the trends in Pten, Syn, Psd95, and C1q expression over time, we performed a Spearman rho R correlation matrix. We find there to be a correlative expression between Pten and Psd95 (*r* = 0.90), as well as inverse correlative expression between Pten and C1q (*r* = 0.70), C1q and Syn (*r* = 0.60), and C1q and Psd95 (*r* = 0.90) (Supplementary Fig. 2a, b). In addition, we find that only at P40 that a strong correlation exists between decreased expression of Pten and decreased expression of the synaptic marker Syn (*r* = 0.94, *p* value = 0.01).

In addition, in *Pten*^*m3m4/m3m4*^ hippocampus, we observe decreases in Syn and Pten expression at the P40 time point relative to *Pten*^*WT/WT*^ controls by Western analysis (Supplementary Fig. 6a, b, c, e). Syn expression on the surface of hippocampal CA3 neurons of *Pten*^*WT/m3m4*^ and *Pten*^*m3m4/m3m4*^ mice actually showed a significant increase in expression by IF analyses during early development at age P14 (Mean Syn: *Pten*^*WT/WT*^ = 1.05 ± 0.13 vs. *Pten*^*m3m4/m3m4*^ = 1.85 ± 0.41, *p* = 0.01 (Fig. 4d, e). However, by P40 Syn expression in the CA3 neurons is significantly decreases in *Pten*^*m3m4/m3m4*^ mice compared with their wild type littermates (Mean Syn: *Pten*^*WT/WT*^ = 0.96 ± 0.33; *Pten*^*m3m4/m3m*^ = 0.47 ± 0.04, *p* value = 0.03) (Fig. 4d, e). To further investigate the increased synapse number in hippocampus at P14 followed by significantly decreased expression at P40, we looked at the presence and activation status of the microglial pruning machinery in the hippocampus. We found that at P14, there is no significant increase in activated microglia (Fig. 4d, e). In contrast at P40, *Pten*^*m3m4/m3m4*^ mice showed a significant increase in microglia activation compared with *Pten*^*WT/WT*^ mice as estimated by activated morphological changes and increased cell area at P40 (Iba1 area: *Pten*^*WT/WT*^ = 3.62 ± 0.89 vs. *Pten*^*m3m4/m3m4*^ = 10.41 ± 1.70, *p* value = 0.001) (Fig. 4d, e), as well as by Iba1 expression levels (relative expression: 1.90 ± 0.44; *F* = 0.7, *p* value = 0.002) (Supplementary Fig. 6d). In addition, C1q expression was also found to be significantly increased in *Pten*^*m3m4/m3m4*^ mice compared with *Pten*^*WT/WT*^ at P40 both in the hippocampus (relative expression:  1.78, *p* value = 0.004) (Supplementary Fig. 6d). Western blot analysis showed no statistically significant increase in C1q expression during earlier developmental time points (Fig. 5g, Supplementary Fig. 1a–e).

These data suggest a correlation in vivo between activation of microglia, increased expression of C1q and decrease in synaptic proteins in the brain, but it is only when the *Pten*^*m3m4/m3m4*^ mouse reaches P40, near its terminal age and coincidentally the nadir of Pten expression do we observe the largest effect.

### *Pten*^*m3m4/m3m4*^ microglia become activation at P21 in mouse cortex

To understand the role of microglial activation and its temporal effect on synapse numbers in the brain, we analyzed microglial activation across development using IF. No significant differences in microglial cell area were observed at P2, P8, or P14 in *Pten*^*WT/m3m4*^ or *Pten*^*m3m4/m3m4*^ mouse cortex compared with wild-type cortex (Fig. 5a–f). Although not statistically significant, several “bushy” microglia were observed at P14. Furthermore, by P21, *Pten*^*WT/m3m4*^ and *Pten*^*m3m4/m3m4*^ mice showed significantly increased microglial activation compared with that of *Pten*^*WT/WT*^ (mean cell area: *Pten*^*WT/WT* ^= 0.51 ± 0.20; *Pten*^*WT/m3m4* ^= 0.59 ± 0.21; *H* = 18.15, *p* value = 0.04; *Pten*^*m3m4/m3m4*^ = 0.72 ± 0.22, *p* value < 0.0001) (Fig. 5e, f). This difference between mutant and wildtype mice was also evident by P40 (mean area: *Pten*^*WT/WT* ^= 0.46 ± 0.20*,  H* = 10.5; *Pten*^*m3m4/m3m4*^ = 0.64 ± 0.29; *H* = 10.5, *p* value = 0.01) (Fig. 5e, f).

Collectively, our in vivo data suggest that dysregulated Pten expression is correlated with changes in microglial activation status and a subsequent decrease in number of synapses at later ages. Although the *Pten*^*m3m4*^ mouse is predisposed to increased synapses consistent with observations in neuron-specific Pten mutant models, the decrease in synapses begins to take place by P21, a correlated time point when Pten levels plunge below 50% of wild-type mice (Fig. 4a). Incidentally, synaptic pruning has been shown to peak in mice also at P21 [24]. We show that dysregulated feedback cues between microglia and neurons involving C1q expression may play some role in the exaggerated decrease in synapses in *Pten*^*m3m4*^ mutants.

## Discussion

This study demonstrates that germline *Pten* mutation that disrupts Pten nuclear localization results in changes in microglial morphology, activation, and function. Using IPA, we re-analyzed the published *Pten*^*m3m4/m3m4*^ neural transcriptome [13], finding enriched networks related to neuroinflammation and synaptic transmission (Fig. 1a, b). We were able to bring these pathway-based predictions to in vitro and in vivo validation. Initially, we utilized a series of co-culture experiments with microglia and neurons from *Pten*^*WT/WT*^ and *Pten*^*m3m4/m3m4*^ genotypes. Our results demonstrate that cytoplasmic-predominant Pten in *Pten*^*m3m4/m3m*^ microglia leads to a greater propensity to prune synapses (Fig. 2), enhanced phagocytic capacity, and increased expression of C1q (Fig. 3). We next confirmed these in vitro findings using the *Pten*^*m3m4/m3m*^ mouse, showing decreased expression of synaptic proteins and increased microglia activity in the cortex and hippocampus (Fig. 4 and Supplementary Fig. 6d). This study is the first to demonstrate a function for Pten in regulating not only microglial activation but also synaptic pruning.

In this study, we demonstrate the first microglial pathology subsequent to a germline *Pten* mutation arising from cell-autonomous mechanisms. Other Pten models have observed similar activated microglial morphologies but fail to pursue any inquiry into Pten function in microglia, making them the last neural cell type requiring Pten function to be characterized [25, 26]. Here, utilizing in vitro culture techniques we were careful to separate the *Pten*^*m3m4*^ microglia from neuronal and glial influences and still demonstrate a microglial pathology (Figs. 2 and 3). Although there is a vast body of literature demonstrating that microglial-dependent synaptopathies are associated with ASD-like behaviors in mice [24], this study does not demonstrate a direct cause–and-effect relationship between our reported microglia pathology and the ASD-like characteristics of the *Pten*^*m3m4*^ model [4, 12, 13]. These observations are purely associative, and it is unknown if the mutant microglia have an impact on neuronal function and the ASD-like phenotypes reported in our model.

Although this study of the effect of *Pten* disruption in microglia is unique, there have been other studies on the effect of *Pten* disruption in myeloid-derived cells, the cellular precursors of microglia. These studies partially corroborate our findings. For instance, when *Pten* is knocked out in myeloid-derived cells (MDCs), they show increased PI3K signaling, elevated G-CSF secretion, and enhanced proliferation [27]. In addition, Pten knockout in MDCs increases phagocytosis of apoptotic cells and differences in cytokine production [28].

Interestingly, there are also some parallels between the microglial phenotypes in the *Pten*^*m3m4*^ model and the microglial phenotypes associated with neurodegenerative disorders, such as Alzheimer’s disease [29, 30]. The activation of microglia and increased synaptic pruning are a feature of many models of neurodegeneration. Collectively, these data suggest that *Pten* functions as a negative regulator of microglial activity in a cell-autonomous fashion. This is manifested most clearly in our in vitro studies of microglia morphology, Iba1 expression, and phagocytosis activity. Additional studies will be required to elucidate the undoubtedly numerous pathways by which Pten disruption contributes to microglial pathology.

In addition, we observed increases in the expression of molecules related to complement and innate immune function: *C1qa-c, C3ar1*, *Cx3cr1,* *Itgam*, *Itgb2*, and *Trem2*. This finding is often reported both in ASD and neurodegenerative models with activated microglia [24, 30, 31] (Table 1). The overexpression of C1q is the most telling of these molecules given its status as a critical mediator of synaptic pruning (Fig. 3a, b). This study is the first reported correlation between Pten and C1q. Thus, our work suggests Pten participates in the regulation of synaptic pruning via complement signaling. Another study associated *Pten* disruption in an MKPOSE model to changes in complement expression, but the genetic complexity of that model previously obfuscated what is now a clear molecular connection [32]. Future studies will be important to dissect out how Pten function regulates C1q expression and determining if there are subsequent physiological consequences relevant to synaptic transmission, neuronal plasticity, and autism behavior.

Here, we show the synaptic pruning by *Pten*^*m3m4/m3m4*^ microglia is due both to cell-autonomous and non-cell-autonomous mechanisms as outlined by our current working model (Supplementary Fig. 7a–c). It is evident that there is an additive effect on pruning from the combination of *Pten*^*m3m4/m3m4*^ neurons with *Pten*^*m3m4/m3m4*^ microglia, illustrated by both the increase in phagocytosis and the increase in C1q foci in these co-cultures (Figs. 2g, and 3c). It is important to note that although we observe decreased nuclear Pten in the brain of the *Pten*^*m3m4*^ mouse, we also observe decreased cytoplasmic Pten [12]. We believe this to be, in part, due to decreased Pten stability, as well as increased proteasomal degradation as a result of the inability of Pten to evade cytoplasmic ubiquitination. In light of this, it is important to note that both decreased stability and nuclear localization have been observed in a subset of human mutations involving Pten from ASD patients [33, 34]. Therefore, the *Pten*^*m3m4*^ mouse is currently the best in vivo mouse model for this type of mutation.

In addition, further work is required to dissect whether the enhanced pruning is because of the increase in dendritic branching (i.e., more “weak” synaptic connections) in the *Pten*^*m3m4/m3m4*^ neurons or because of an unknown “eat me” signal [14, 15]. As discussed earlier, we observed that *Pten*^*m3m4/m3m4*^ neurons cultured in vitro have more synapses compared with *Pten*^*WT/WT*^ and *Pten*^*WT/m3m4*^ (Supplementary Fig. 4a, b). These data are supported by the work of others who have shown *Pten* deletion in neurons results in increased synaptic spine density and dendritic arborization [35–37].

Interestingly*,* in vivo, we observe a significant decrease in Syn expression at P21, and by P40, this effect is even greater with significantly decreased Psd-95 expression (Fig. 4b, c). While in vitro, we see significant decreases in synapses at DIV 14 when co-cultured with microglia for 7 days. Perhaps these subtle differences in the timing of decreased synapses may be due to the microenvironment of our neuron-microglia co-culture system, which may not be as complex as the in vivo context [38, 39]. These difference in the timing of synaptic loss may also be partially due to a lack of the milieu of exogenous cellular factors, which also must undergo temporal changes, that would only be present in vivo. The literature has demonstrated that synaptic pruning peaks in mice at P21 [15], when we first see significantly decreased Syn expression and increased microglia activation in the *Pten*^*m3m4/m3m4*^ model (Figs. 4b and 5a, e). We would like to note, that although there is not a significant change in microglia morphology in the *Pten*^*m3m4/m3m4*^ until P21, we did observe some microglia activated in the cortex at P14, which is concordant with synaptic pruning in our DIV 14 neuron-microglia co-cultures (Fig. 5a). Despite the primary neuron cultures only having 7 days to incubate with microglia, they show signs of significant synaptic pruning already, suggesting that pruning occurs earlier in vitro than in vivo. This could be explained by one of two non-mutually exclusive contexts: (1) priming of primary microglia due to culture conditions prior to isolation and incubation with primary neurons; or (2) exogenous regulators (other cells and their signals) that are lacking in the in vitro system. The direct mechanism of how synaptic pruning in microglia is initiated by P21 and subsequently repressed remains unclear, however our data suggest that Pten signaling may be a key regulator of this process.

Another avenue to consider in this study and moving forward is that the *Pten*^*m3m4*^ model is not a conventional “knockout”, rather it is a “knock-in” germline mutant. This is an innovative aspect to our study since the germline model more accurately recapitulates the systemic effects of Pten dysfunction observed in our *PTEN* germline mutation-positive patients with ASD. It would be interesting to investigate other human-PTEN-ASD mimicking mutant mouse models with respect to Pten structure, function, subcellular localization, and overall phenotype, including the effects on neurons, astrocytes, oligodendrocytes, and microglia. Perhaps some mutations would present in a “spectrum” of minor to severe phenotypes as described in clinical cases of ASD with respect to behavior, and perhaps even to cellular severity of the phenotype.

This study identifies a new role for Pten in influencing microglia activity, especially in the context of synaptic pruning, a normal neurodevelopmental process that supports a healthy synaptic architecture. Although this study is limited in that it cannot cleanly distinguish between the effects of cell-autonomous and exogenous factors on synaptic pruning, it identifies an important role for Pten in microglia, where Pten acts to guard against neuroinflammation, preventing aberrant activation and enhanced phagocytosis. Moreover, this observed microglia pathology may be relevant to neurodevelopmental and synaptopathic phenotypes such as autism.

## Supplementary information

Supplemental Materials
