# Supplementary material for: Cytoplasmic-predominant Pten increases microglial activation and synaptic pruning in a murine model with autism-like phenotype
Source: Mol Psychiatry. 2020 Feb 13;26(5):1458–71. doi: 10.1038/s41380-020-0681-0 (PMC8159731; doi:10.1038/s41380-020-0681-0)
Supplement: Supplementary file 1 — Supplemental Materials [file 41380_2020_681_MOESM1_ESM.pdf]

Supplementary Figure 1

A

P40 CX

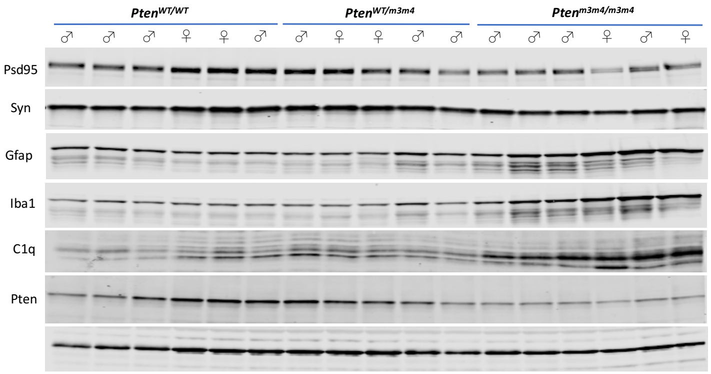

B

P21 CX

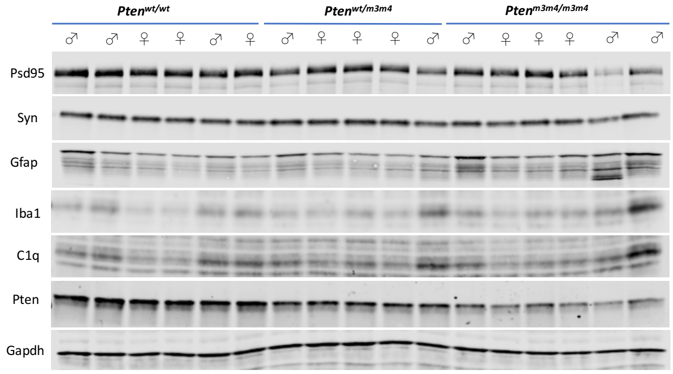

C

P14 CX

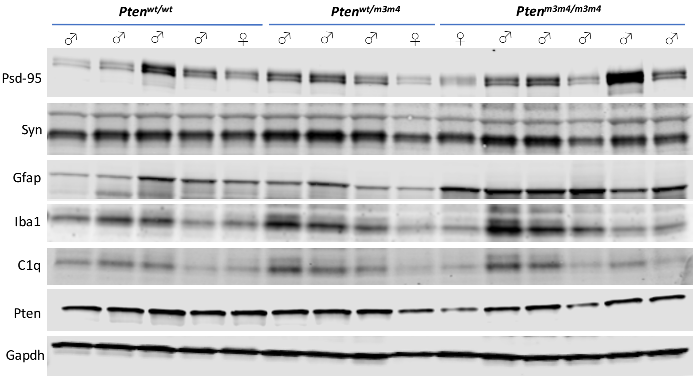

D

P8 HB

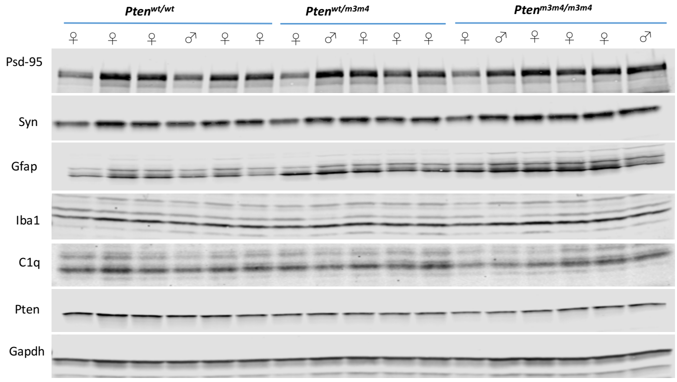

E

P2 HB

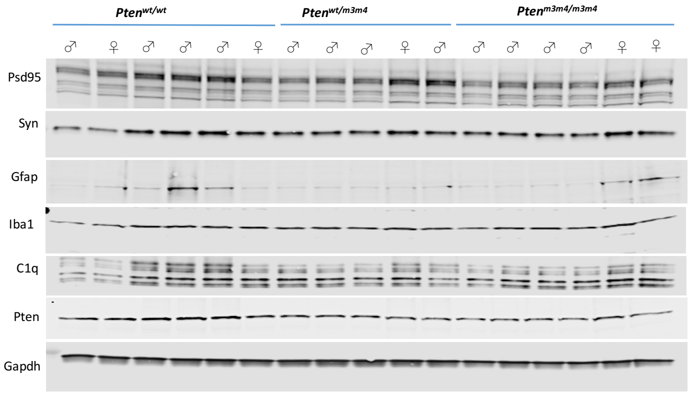

Supplementary Figure 1: Time point Western blot data for *Pten*$^{WT/WT}$, *Pten*$^{WT/m3m4}$, and *Pten*$^{m3m4/m3m4}$ cortex and Hemi-brain. (a-c) P40, P21, and P14 cortical Western blot data showing expression of Psd95, Syn, Gfap, Iba1, C1q, Pten, and Gapdh with genders of each biological replicate. (d and e) Hemi-brain Western plots of P8 and P2, showing expression of Psd95, Syn, Gfap, Iba1, C1q, Pten, and Gapdh with corresponding genres.

Supplementary Figure 2

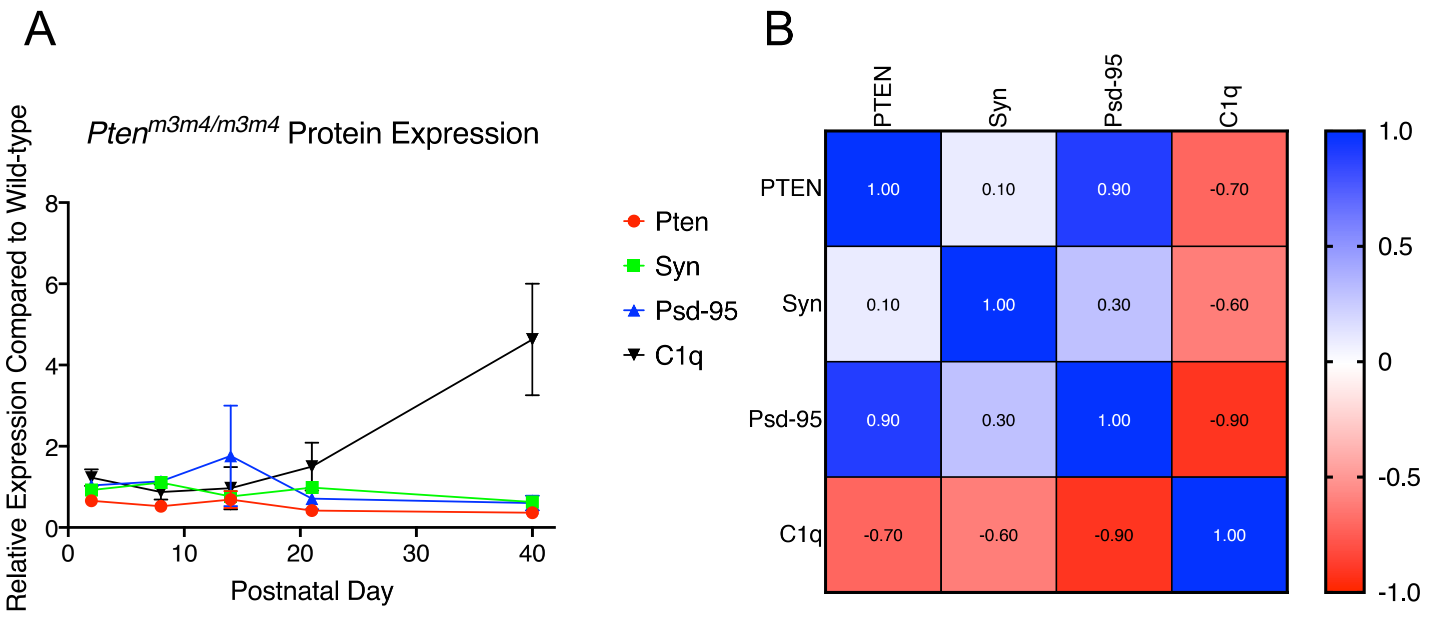

Supplementary Figure 2: Correlative analysis of *Pten*$^{m3m4/m3m4}$ expression of Pten, Syn, Psd95, and C1q. (a) Cortical Western blot data of *Pten*$^{m3m4/m3m4}$ plotted over time for Pten, Syn, Psd95, and C1q. (b) Spearman rho R correlation matrix depicting positive and negative correlations for proteins plotted in supplemental figure 6a in the *Pten*$^{m3m4/m3m4}$ mouse. These data show a corelative expression between Pten and Psd95 (r = 0.90, p-value = 0.083), as well as inverse correlative expression between Pten and C1q (r = 0.70, p-value = 0.23), C1q and Syn (r = 0.60, p-value = 0.35), and C1q and Psd95 (r = 0.90, p-value 0.83).

Supplementary Figure 3:

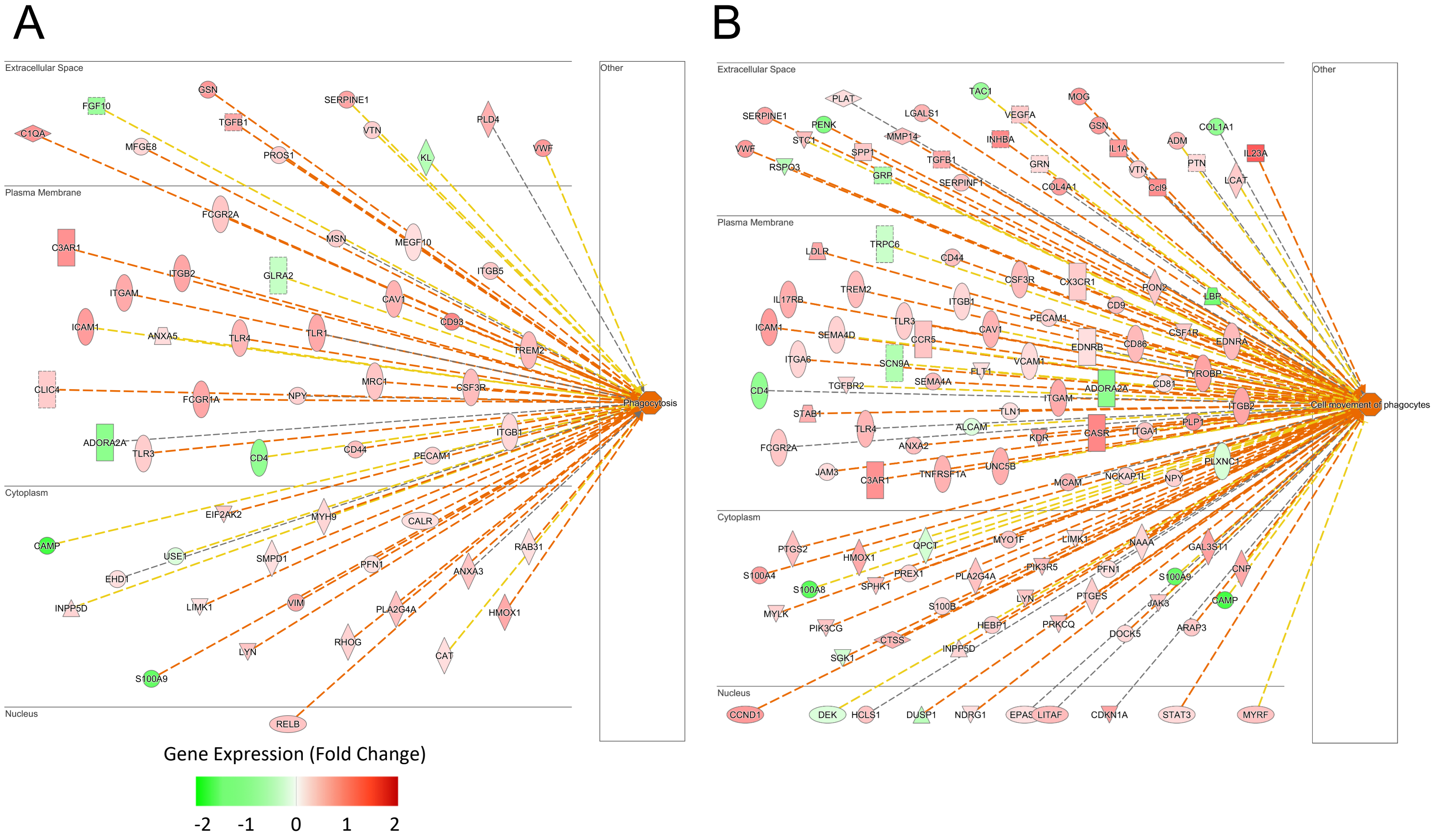

Supplementary Figure 3: IPA network analysis of *Pten*$^{m3m4/m3m4}$ P40 cortical transcriptome shows increased activation of pathways pertaining to phagocytosis and cell movement of phagocytes. (a) IPA network analysis clustered 57 genes related to phagocytosis, p-value = 1.19E-10; z-score = 2.98. (b) IPA network analysis clustered 118 genes related to movement of phagocytes, p-value = 1.37E-23; z-score=4.46.

Supplementary Figure 4

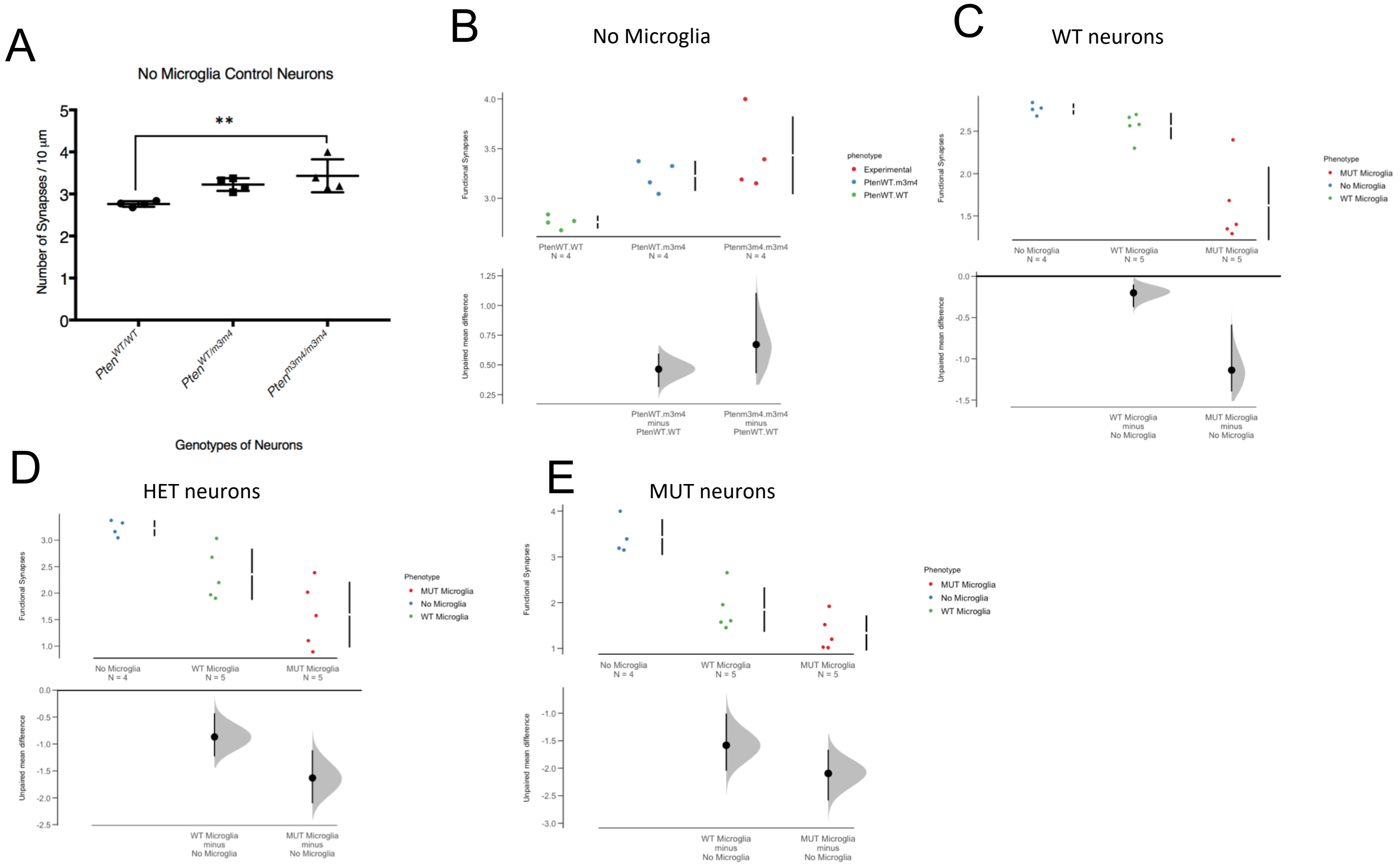

Supplementary Figure 4: Primary neuron control data and estimation plots of neuron-microglia co-culture experiments. (a) Number of synapses quantified from primary neuron cultures from *Pten*$^{WT/WT}$, *Pten*$^{WT/m3m4}$, and *Pten*$^{m3m4/m3m4}$. (b-e) Estimation plots of microglial-neuron co-culture experiments plotting effect size between no microglia, wild-type microglia, and mutant microglia with corresponding neuronal genotypes.

Supplementary Figure 5

A

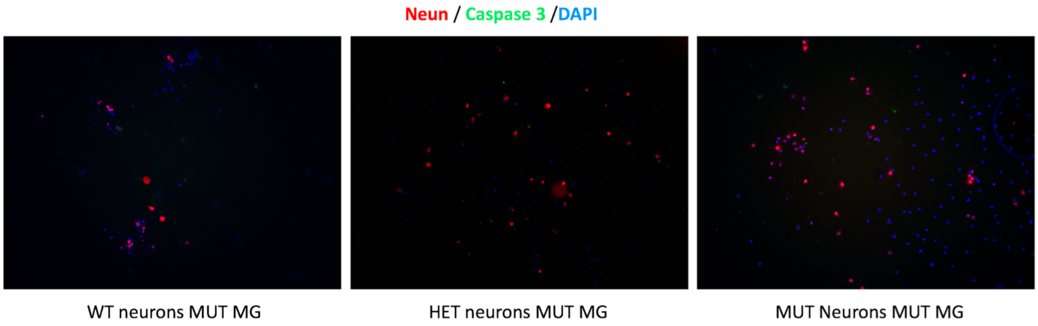

B

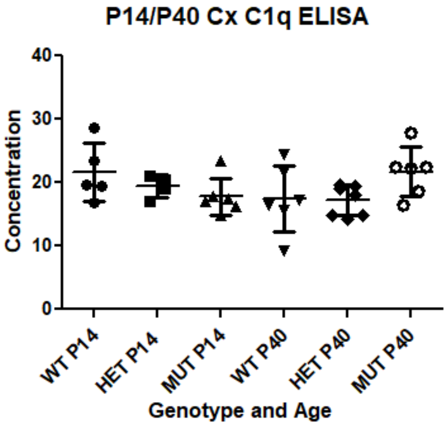

Supplementary Figure 5: Apoptosis assay for neurons co-cultured with mutant microglia and preliminary ELISA quantification of C1q expression. (a) Primary neurons labeled with neun (red) cultured with *Pten*$^{m3m4/m3m4}$ microglia and stained for apoptotic marker Caspase 3 shows no increase in apoptosis. (b) C1q ELISA of P14 and P40 cortical lysate from *Pten*$^{WT/WT}$, *Pten*$^{WT/m3m4}$, and *Pten*$^{m3m4/m3m4}$.

Supplementary Figure 6

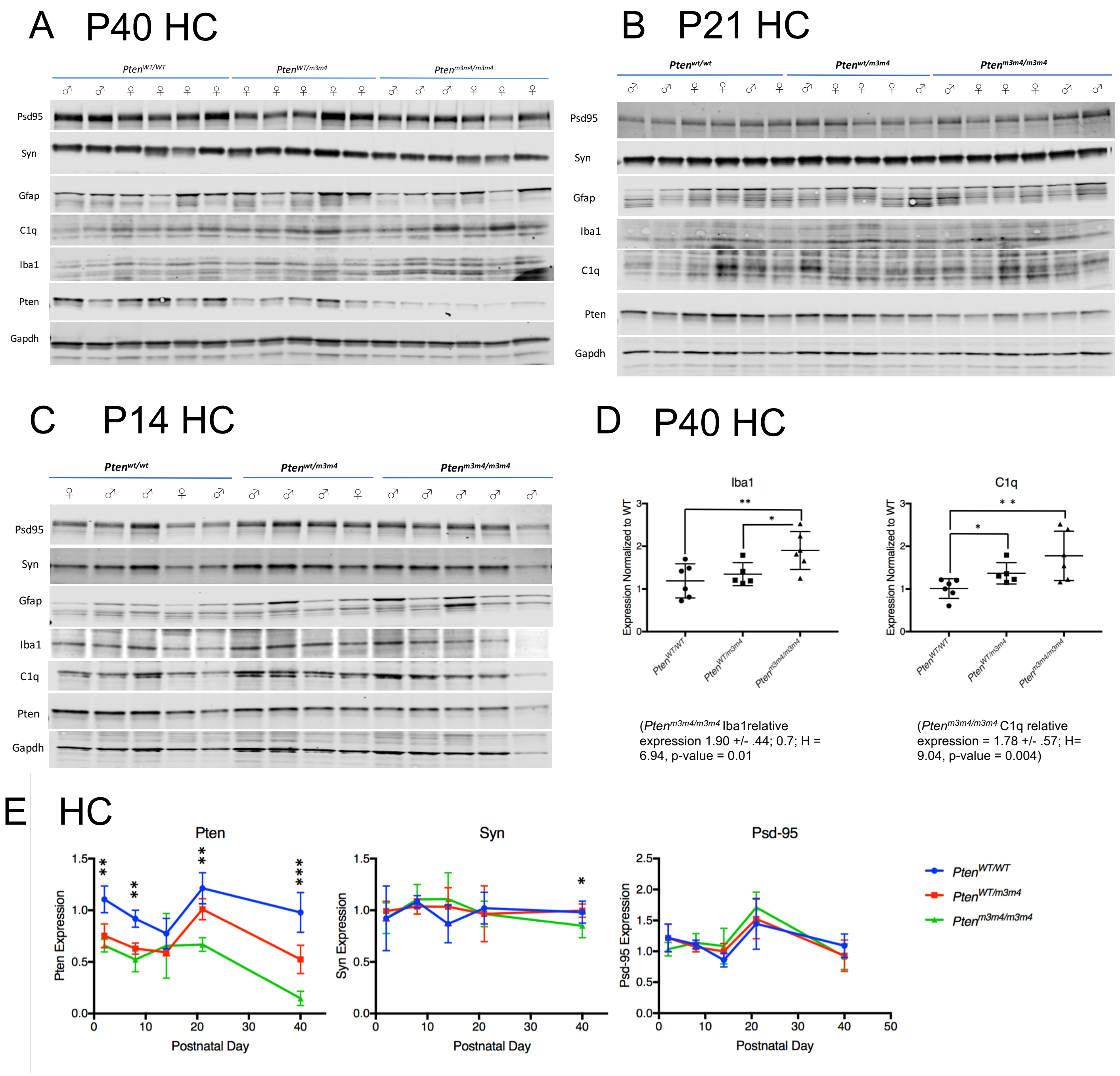

Supplementary Figure 6: Time point Western bot data for $\textit{Pten}^{WT/WT}$, $\textit{Pten}^{WT/m3m4}$, and $\textit{Pten}^{m3m4/m3m4}$ hippocampus. (a-c) P40, P21, and P14 hippocampal Western blot data showing expression of Psd95, Syn, Gfap, Iba1, C1q, Pten, and Gapdh with genders of each biological replicate. (d) Quantification of P40 Western blot data for Iba1 and C1q. (e) Hemi-brain Western blot data plotted with hippocampus Western blot data showing expression of Pten, Sn, and Psd95 at P2, 8, 14, 21, and 40.

Supplementary Figure 7

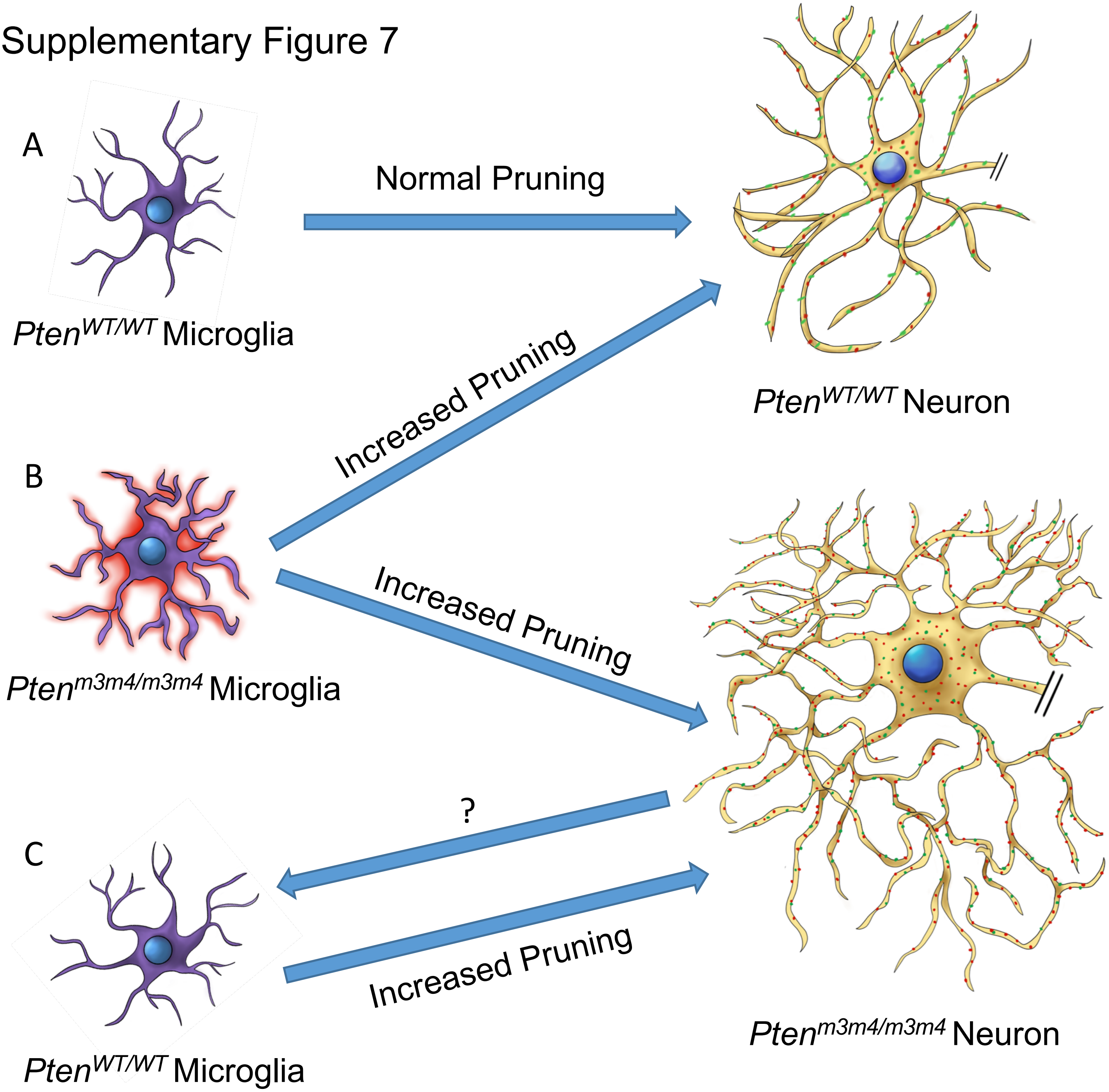

Supplementary Figure 7: Schematic of proposed hypothesis that nuclear Pten act as a negative regulator of synaptic pruning in both microglia and neurons. (a) Normal pruning occurs between wild-type microglia and neurons in co-culture. (b) Decreased nuclear localization of Pten in microglia results in increased synaptic pruning in neuronal-co-culture irrespective of neuronal genotype. (c) Wild-type microglia co-cultured in the presence of neurons deficient in nuclear Pten show increased synaptic pruning efficiency.

Supplementary Table 1

Immune related genes

Gene ID	Fold Change	P-value	Q-value
Cd180	4.167926973	0.00015	0.004911
Il23a	3.854970981	0.00185	0.034578
Ppp1cc	3.681924388	5.00E-05	0.001914
Cd84	2.416602185	5.00E-05	0.001914
Ptgs1	2.292878646	5.00E-05	0.001914
Gbp4	1.90867438	0.00205	0.037434
Ldlrap1	1.81698058	5.00E-05	0.001914
Litaf	1.74108061	5.00E-05	0.001914
Ptgs2	1.704973127	5.00E-05	0.001914
Plekhg2	1.674415997	0.00125	0.025612
Plekhg1	1.667040322	5.00E-05	0.001914
Cryab	1.657825317	5.00E-05	0.001914
H2-T10	1.568050475	0.0001	0.003528
H2-K1	1.559558857	0.0001	0.003528
Dab2	1.551906887	0.00055	0.013915
Tril	1.530419329	5.00E-05	0.001914
Plekhg3	1.516742313	5.00E-05	0.001914
Cebpa	1.479137325	0.001	0.021638
Acss2	1.439063247	5.00E-05	0.001914
Gab1	1.429199189	5.00E-05	0.001914
H2-D1	1.425226289	5.00E-05	0.001914
Acer2	1.397524912	0.00055	0.013915
Stat3	1.364895974	0.0002	0.006208
Hagh	1.360826367	0.0002	0.006208
Tns3	1.356767954	0.0001	0.003528
Hepacam	1.348897757	0.00015	0.004911
Acsbg1	1.318117517	0.0003	0.008597
Tns1	1.302916835	0.00285	0.047438
Mapk8ip1	1.259459735	0.0025	0.043297
Lmo3	-1.635569426	5.00E-05	0.001914
Psg16	-1.762048946	0.0009	0.019989
Lbp	-3.078996015	5.00E-05	0.001914

(GO: 0002376)

Supplementary Table 2

Synaptic function related genes

Gene ID	Fold Change	P-Value	Q-Value
P2rx1	2.314531183	0.00115	0.02398
Bdnf	2.241089088	0.00005	0.001914
Mctp2	1.869969838	5.00E-05	0.001914
Syngr2	1.585761715	0.00065	0.01563
Syt2	1.428420753	0.00005	0.001914
Grin2c	1.341073213	0.00055	0.013915
Syn2	1.319206123	0.0005	0.012867
Grik3	-1.263589628	0.002	0.036706
Unc13c	-1.319670722	0.00035	0.009729
Syt17	-1.348214456	0.002	0.036706
Mctp1	-1.364835427	5.00E-05	0.001914
Shisa9	-1.37439815	0.0002	0.006208
Syt4	-1.436686216	0.00005	0.001914
Grik2	-1.446794481	5.00E-05	0.001914
Glra2	-1.586815066	0.00005	0.001914
Calb2	-1.995373749	0.00005	0.001914

(GO: 0045202)

Supplementary Table 3

Control Neurons				
P- Value Summary = **	P - Value	Mean Diff.	DF	F = 11
$Pten^{WT/WT}$ vs. $Pten^{WT/m3m4}$	0.06	-1.1	9	
$Pten^{WT/WT}$ vs. $Pten^{m3m4/m3m4}$	0.0095	-1.5	9	
$Pten^{WT/m3m4}$ vs. $Pten^{m3m4/m3m4}$	0.49	-0.45	9	
WT Neurons				
P- Value Summary = ***	P - Value	Mean Diff.	DF	F = 20
No Microglia vs. $Pten^{WT/WT}$ Microglia	0.39	0.2	11	
No Microglia vs. $Pten^{m3m4/m3m4}$Microglia	0.0038	1.1	11	
$Pten^{WT/WT}$ Microglia vs. $Pten^{m3m4/m3m4}$Microglia	0.21	0.94	11	
HET Neurons				
P- Value Summary = **	P - Value	Mean Diff.	DF	F = 13
No Microglia vs. $Pten^{WT/WT}$ Microglia	0.051	0.87	11	
No Microglia vs. $Pten^{m3m4/m3m4}$Microglia	0.001	1.6	11	
$Pten^{WT/WT}$ Microglia vs. $Pten^{m3m4/m3m4}$Microglia	0.07	0.76	11	
MUT Neurons				
P- Value Summary = ****	P - Value	Mean Diff.	DF	F = 28
No Microglia vs. $Pten^{WT/WT}$ Microglia	0.0005	1.6	11	
No Microglia vs. $Pten^{m3m4/m3m4}$Microglia	<0.0001	2.1	11	
$Pten^{WT/WT}$ Microglia vs. $Pten^{m3m4/m3m4}$Microglia	0.19	0.51	11	

Supplementary Table 4

Syn Co-localized in Microglia		
P value summary = ****	Summary	P Value
WT MG / WT Neur vs. MUT MG / WT Neur	*	0.04
WT MG / WT Neur vs. MUT MG / HET Neur	****	<0.0001
WT MG / WT Neur vs. MUT MG / MUT Neur	****	<0.0001
WT MG / HET Neur vs. MUT MG / MUT Neur	***	0.0009
WT MG / MUT Neur vs. MUT MG / MUT Neur	*	0.02
